# Development of a Piezoceramic Harvester for Sea Waves Energy Recovery in Environmental Monitoring Buoys

**DOI:** 10.3390/s25072046

**Published:** 2025-03-25

**Authors:** Roberto Montanini, Antonio Cannuli, Fabrizio Freni, Antonino Quattrocchi, Andrea Venuto

**Affiliations:** Department of Engineering, University of Messina, C.da di Dio, 98166 Messina, Italy; roberto.montanini@unime.it (R.M.); antonio.cannuli@unime.it (A.C.); antonino.quattrocchi@unime.it (A.Q.); andrea.venuto@unime.it (A.V.)

**Keywords:** sea wave energy recovery, energy harvester, piezoceramic devices, electromechanical characterization

## Abstract

In the last decades, marine environment monitoring has gained significant attention as it plays a fundamental role in ecosystem health and anthropogenic impact evaluation. This study presents the development of a sea wave energy recovery device based on piezoceramic harvesting, designed to contribute to the energy self-sufficiency of an environmental monitoring buoy. The system consists of a flexible S-shaped arm anchored to the buoy structure; the buoyancy system at the free end converts wave-induced motion into mechanical stress, deforming the opposite side of the arm, where piezoceramic patches are installed to generate electrical power. An extensive experimental campaign was conducted to perform the electromechanical characterization of the device and to analyze the manufacturing quality of the arm, produced by stereolithographic additive manufacturing. The results demonstrate the ability to harvest kinetic energy across a range of wave frequencies and amplitudes. Under the best conditions, a maximum transfer electric power of 220.2 ± 3.7 µW was reached.

## 1. Introduction

In recent decades, marine environment monitoring has gained significant attention within the scientific and technological community. This activity plays a fundamental role in the quantitative evaluation of ecosystem health and anthropogenic impacts, often relying on advanced multiparameter statistical forecasting models [1,2]. Key applications include recording meteoclimatic phenomena, measuring water salinity, assessing seismic safety in coastal areas, and monitoring pollutants dissolved or suspended in water [3,4,5]. These efforts generate extensive datasets that can be historicized and geolocated, providing valuable insights for environmental science, engineering, and resource management [6]. Measurement buoys represent a cornerstone of marine monitoring systems, allowing the integration of various sensors and technologies for data acquisition, storage, and real-time transmission. However, despite advancements in low-power electronics that have significantly reduced energy consumption, energy autonomy and supply remain major challenges for these devices, particularly to allow applications in remote and inaccessible locations where regular maintenance is impractical.

Traditional solutions, such as photovoltaic and wind energy systems [7,8], are widely adopted but are subject to inherent limitations. Solar panels rely on the day–night cycle, while wind turbines require favorable meteorological conditions. Both approaches often require large battery storage systems to ensure continuous power supply, adding weight and complexity to buoy deployment and operation. In this context, alternative solutions based on energy harvesting leveraging the continuous and predictable motion of sea waves have gained increasing interest. A considerable number of systems for energy harvesting with nearshore buoy application have been documented in the literature, differentiating from each other based on the energy conversion system position (internal vs. external), the operating principle (overtopping, oscillating water column, oscillating body systems) and the relative movements (parallel or series) [9]. The main systems include floating body mechanisms exploiting the structure of the buoy [10], turbine mechanisms that involve the implementation of one or more turbines [11], pendulum-based mechanisms that rely on the oscillation of a buoy rod [12], and mechanisms based on mass-spring couplings characterized by simplicity of construction [13].

Among the alternative solutions, piezoelectric energy harvesting has emerged as a promising candidate due to its simplicity and reliability [14]. Piezoelectric energy harvesters can employ either bulk piezoelectric disks (PZTs) or piezoceramic patches (PCPs). The latter typically consist of a multilayer structure made of a thin zirconated lead titanate (PbZrTiO_3_) film sandwiched between two electrodes and encased in a protective polymeric shell. When subjected to mechanical deformation, PCPs generate an electrical charge proportional to the applied strain amplitude and frequency [15,16]. In conventional applications, piezoceramic energy harvesters are designed to operate at mechanical resonance conditions [17], where they achieve the maximum energy conversion efficiency and significant power outputs, sometimes reaching several tens of milliwatts [18,19]. This approach is effective in controlled environments with well-defined forcing excitations, which is not the case in marine conditions, where wave motion is characterized by inherent variability in both amplitude and frequency. This necessitates the development of energy harvesting systems capable of operating effectively outside resonance conditions, prioritizing continuous modest power generation rather than peak efficiency. Additionally, the low-frequency nature of sea waves enables the use of simplified harvester designs due to reduced mechanical stress, potentially simplifying the extension of their operational lifespan [20].

Several studies have investigated the potential of different piezoelectric configurations for sea wave energy harvesting. Wu et al. [21] developed a floating 20-m-long buoy anchored to the ocean floor, equipped with piezoelectric cantilevers with a length of 1 m, capable of generating up to 24 W of electrical power. Kazemi et al. [22] developed an immersible and waterproof wave energy harvester with piezoelectric cantilevers to capture energy from the longitudinal and lateral motion of waves, generating a maximum power density of 158 mW/m^2^. Salazar et al. [23] proposed a body caudal fin energy harvester, in which a piezoelectric patch was attached to the tail end. The functionality of the harvester was based on the impact of waves, causing deformation of the piezoelectric plate at the end, thereby generating electricity. Alizzio et al. [24] proposed a fixed-point buoy with deformable floating appendages, where PCPs were bonded to function as rolling deformable bars. Du et al. [25] designed an innovative rack-and-pinion mechanism to convert the sea wave motion into a cam with unidirectional rotation, driving a multi-pillar piezoelectric stack.

In such applications, the low mechanical and electrical conversion efficiency and the limited output power related to the low frequency of sea waves can affect the potential development of marine wave direct-coupled PEH devices [26]. In addition, in most of these cases, PCPs are used without exploiting mechanical resonance, and for this reason, a system design optimization is needed to maximize the energy conversion performance [27].

All the abovementioned solutions highlight the feasibility of using piezoelectric technology in marine environments, focusing attention on subsurface installations to capture transversal shallow ocean waves. As monitoring buoys are often designed to operate in protected nearshore environments with low-energy waves, a mechanical system able to unlock the buoy pitching motion potential must be studied.

This paper focuses on the development of a piezoceramic energy harvester designed for integration into a measurement buoy. The system operates through a dynamic inertial behavior designed to work in phase opposition to the buoy waterline motion and consists of a resin S-shaped arm molded by a stereolithographic printer, that can be easily attached to the buoy body. Two piezoceramic patches are installed near the arm fixed end, while a floater is connected to the free end to transfer wave-induced motion. The arm shape and dimensions are designed to maximize mechanical deformation under wave-induced motion, ensuring efficient kinetic energy transfer to the PCPs. This simple, yet effective, design allows the energy harvester to operate across a range of wave amplitudes and frequencies. The mechanical and electrical performance of the device was evaluated experimentally under controlled conditions, simulating realistic sea wave dynamics for nearshore applications. Key parameters such as frequency, amplitude, and mechanical strain were systematically varied to assess their impact on the generated voltage and transferred effective power. Preliminary results indicate that the proposed device can generate sufficient energy to power small onboard sensors in a standalone configuration. In addition, it differs from solutions in the literature since it can be easily installed on pre-existing buoys, even in limited overall available space. Finally, the modular nature of the system allows for the installation of multiple devices in a single buoy, providing a step toward energy self-sufficiency in marine monitoring applications.

The research was conducted as part of the TETI (Innovative Technologies for Control, Monitoring and Safety at Sea) project funded by the Italian Operational Program (PON Ricerca e Innovazione 2014–2020). In addition, the activity was partially funded by European Union’s Next Generation EU, project MUR, PNRR-M4C2, ECS_00000022—SAMOTHRACE (Sicilian Micro and Nano Technology Research and Innovation Center).

## 2. Materials and Methods

### 2.1. Energy Harvesting System

**Figure 1:**
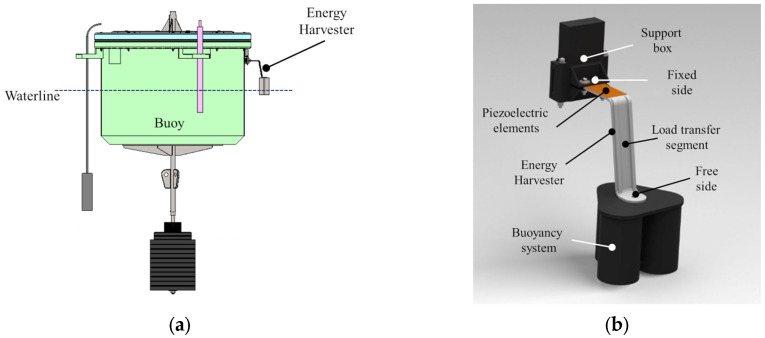
(**a**) 3D rendering of the buoy system with the energy harvesting device installed on the body and (**b**) the detailed scheme of the proposed device, where the main components can be identified: support box, S-shaped arm with PCPs bonded, buoyancy system.

An innovative energy harvester was developed to convert the kinetic energy of sea waves into electrical energy, exploiting the electrical hysteresis properties of piezoceramic materials. The system, shown in Figure 1a, operates through a dynamic inertial behavior designed to work in phase opposition to the buoy waterline motion, ensuring efficient energy transfer. In order to allow for the potential installation on the body of already existing buoys, the configuration of the device, shown in Figure 1b, consists of a mechanically rigid S-shaped arm, aimed at transferring the wave-induced motion to PCPs.

The S-shaped arm is composed of three distinct functional sections. At the horizontal fixed end rigidly connected to the body of the buoy, a housing was designed to accommodate two PCPs placed on the upper and lower surfaces. This section serves as active element, where the mechanical strain induced by wave motion is converted into electrical energy. A vertical load transfer segment connects the fixed end to the free end, acting as a structural intermediary transmitting kinetic energy without significant self-deformation and energy dissipation. The horizontal free end, positioned near the waterline, supports a cylindrical buoyancy system designed to harness the pitching motion of the buoy induced by wave activity. This system, half-immersed in equilibrium, translates the kinetic energy of the buoy into cyclic deformation of the arm, enabling continuous energy generation.

**Figure 2:**
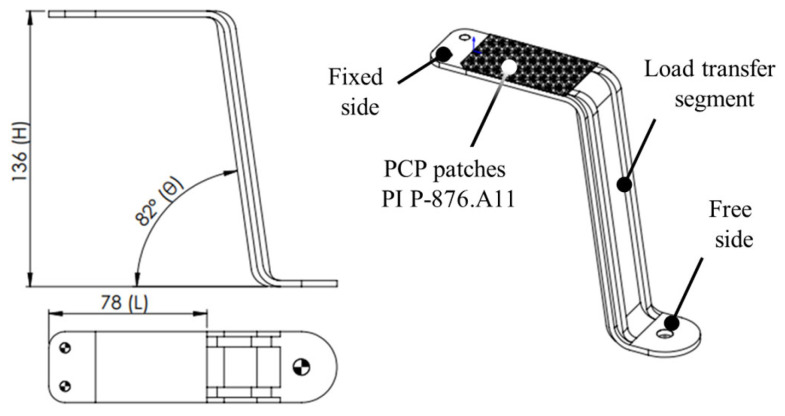
Detailed design of the S-shaped arm with the main geometrical design parameters: H—neutral plane height, L—patch housing extension, θ—heeling angle.

In order to enhance the device energy conversion capacity, the main requirement is to maximize the PCPs housing section strain, avoiding unnecessary deformations of the other functional sections of the S-shaped arm (i.e., the vertical load transfer segment and the free end). A finite element model (FEM) was, therefore performed with the aim of optimizing the vertical load transfer segment stiffness and the arm geometrical design parameters: H—neutral plane height, L—patch housing extension, θ—heeling angle (Figure 2).

The FEM model mesh, comprising 38,374 tetrahedral elements of 2 mm in size, was defined through convergence analysis to guarantee optimal computational time and results reliability. The simulation material properties were customized based on the real material (resin black V4) used for the experimental work, as reported in Table 1. The system was simulated assuming a fixed constraint in the arm fixed end, and a vertical force was applied to the arm free end. The model helped in the definition of the reinforcing ribs in the vertical segment, which mitigate energy dissipation, ensuring efficient load transfer in the PCP housing area. A sensitivity analysis was conducted by varying the geometrical design parameters without exceeding the maximum device volume (i.e., limitations in the manufacturing process), allowing for the selection of the optimal arm dimensions to maximize the average deformation of the PCP housing area.

The arm was fabricated by additive manufacturing technique, employing a stereolithographic printer Form 2 (Formlabs, Somerville, MA, USA) with a resolution of 0.05 mm per layer to achieve high-precision fabrication, producing a total of 775 layers. To tailor the mechanical properties of the arm for optimal energy conversion performance, two versions were produced using two photopolymer resins with different characteristics: Black V4 (Formlabs) with moderate stiffness and flexibility and Rigid 4000 (Formlabs) with higher stiffness and enhanced mechanical resistance as shown in Table 1.

Upon completion of the printing process, the post-processing steps included washing in isopropyl alcohol for 20 min to remove residual material and curing in an ultraviolet chamber (Formcure, Formlabs) under specific conditions recommended by the manufacturer: 30 min at 60 °C for the Black V4 resin and 15 min at 80 °C for the Rigid 4000 resin.

The PCPs selected for this application were DuraAct P876.A11 (Physik Instrumente GmbH, Karlsruhe, Germany), consisting of a modified lead zirconate titanate (PIC255) active element encased in a protective Kapton layer, ensuring durability even in challenging marine environments. The patches, with dimensions of 61.0 mm × 35.0 mm × 0.5 mm, are characterized by a charge coefficient *d_31_* of −180 pC/N and an electrical capacitance of 90 nF ± 20% and are capable of operating across a wide temperature range (from −20 °C to 150 °C), ensuring consistent performance under varying environmental conditions. The installation of the patches involved bonding them to the upper and lower faces of the arm housing section using a cyanoacrylate adhesive. To ensure efficient mechanical stress transfer, the adhesive was evenly distributed across the surfaces and side edges of the patches, with complete curing achieved after a 24 h period in static air, as recommended by the manufacturer.

### 2.2. Simplified Mathematical Model

In operating conditions, the buoyancy system connected to the arm undergoes vertical motion driven by the pitching of the buoy. The system consists of a foam cylinder with a diameter *D_bs_* = 0.16 m and a length *L_bs_* = 0.20 m, positioned at a radial distance of *R_b_* = 0.60 m from the buoy vertical axis of rotation. These dimensions were defined in order to avoid excessive forces acting on the arm free end, which could jeopardize the PCP-arm structural integrity.

To model the forces acting on the energy harvester, the hydrodynamic interactions between the buoyancy system and the surrounding water were considered, assuming a periodic buoy motion in the same plane as the arm. The total force transferred to the arm results from the combined effect of three main components: buoyancy, hydrodynamic drag, and viscous shear forces.

In equilibrium conditions, when the buoy is in its neutral vertical position, the cylindrical floater is partially submerged, with an immersion depth of half its length (*L_bs,imm_* = 0.1 m), generating an initial buoyancy force of 19.7 N upward (+Z). As the buoy oscillates, the floater undergoes periodic immersion or emersion, leading to a time-dependent variation in the buoyancy force. The floater is fully submerged when the buoy reaches a pitching angle of *θ_max_* = 9.6 °, corresponding to a maximum buoyancy force of 39.4 N (+Z), while full emergence occurs at *θ_min_* = –9.6 °, leading to a buoyancy force of 0 N.

The hydrodynamic drag [29] acts on the circular faces of the floater as it moves vertically due to the buoy angular oscillation, which was assumed to range within the *θ_min_* to *θ_max_* interval. In general, the drag force opposes the motion: during immersion in water (–Z), the drag force acts upward (+Z), while during emersion in air (+Z) the drag force acts downward (–Z). The resulting force can be described using the standard drag equation:

(1) $$F_{D}=\frac{1}{2}\cdot C_{D}\cdot \rho \cdot A_{t}\cdot v_{v}^{2}$$

where *C_D_* = 1.12 is the drag coefficient for a normal incidence circular plate, *ρ* is the fluid density (1000 kg/m^3^ for water and 1.225 kg/m^3^ for air), *A_t_* = 0.020 m^2^ is the transversal area of the floater, and *v_v_* is the vertical velocity induced by the buoy angular motion, that can be expressed as $R_{b}\cdot \theta_{max}\cdot \omega \cdot \cos\left(2\pi ft\right)$, where *f* is the oscillation frequency of the buoy. Considering the typical nearshore conditions where the wave frequency ranges from 1 to 2 Hz [30], this drag force reaches the maximum value of 4.5 ÷ 17.9 N during immersion (+Z) while remaining negligible during emersion (–Z). The drag force is phase-shifted by 90° with respect to the buoyancy force.

The viscous shear forces act along the lateral walls of the floater, opposing its vertical motion relative to the surrounding fluid. This contribution can be estimated using the following relationship:

(2) $$F_{v}=3\cdot \pi \cdot \mu \cdot D_{bs}\cdot v_{v}$$

where *μ* = 1.002 × 10^−3^ Pa · s is the dynamic viscosity of water. Given the velocity range of the floater, the maximum contribution of viscous shear forces is on the order of ±0.3 N, making it negligible compared to buoyancy and drag forces.

**Figure 3:**
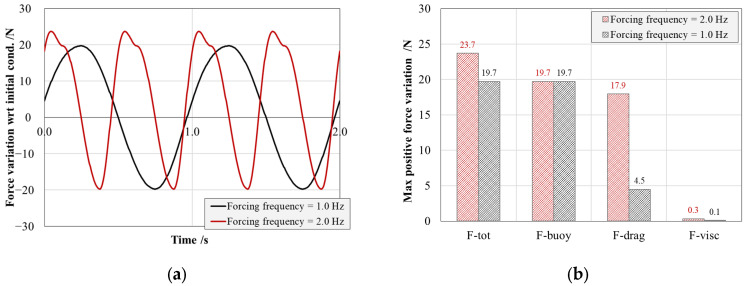
(**a**) Temporal evolution of the force variation acting on the energy harvester arm with respect to the initial condition in the case of buoy oscillation in the interval from 9.6° to −9.6° with the frequency of 1 and 2 Hz. (**b**) Focus on the different force contributions in the two frequency scenarios.

Since piezoceramic active elements generate electrical power as a result of instantaneous deformation, it is crucial to analyze the total force evolution with respect to the initial equilibrium condition, where an initial load is already present due to a portion of the buoyancy force. Figure 3a shows the instantaneous variation with respect to the initial condition of the total force acting on the arm during sinusoidal pitching of the buoy at 1.0 and 2.0 Hz. It becomes clear that the total resulting force is buoyancy dominated at lower frequencies tending to increase with the pitching frequency as the vertical velocity of the floater, which directly impacts the drag force component, as shown in Figure 3b. In the assumed conditions, the maximum forces acting on the arm are equal to 19.7 N and 23.7 N in the case of 1 and 2 Hz pitching, respectively.

### 2.3. Analysis and Characterization

The experimental investigation of the energy harvesting system primarily focused on assessing the electromechanical characteristics of the complete arm–PCP assembly device and the fabrication quality of the S-shaped arms.

**Figure 4:**
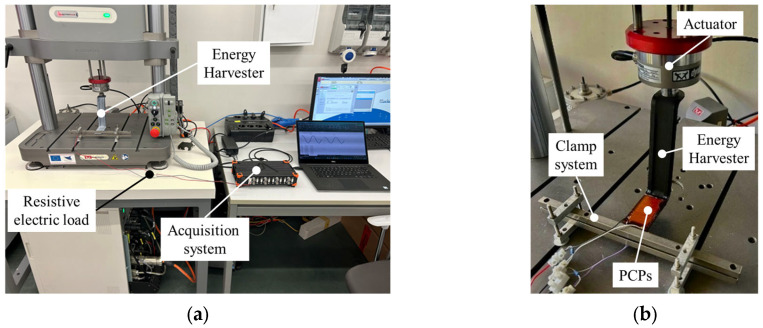
(**a**) Experimental setup used for electromechanical characterization and (**b**) a detailed depiction of the arm connection with the electrodynamic testing machine actuator.

To evaluate the electromechanical behavior of the arm–PCP system and verify its potential performance, a specific experimental setup was developed. The setup, shown in Figure 4a, was composed of an electrodynamic testing machine (model ElectroPuls E3000, Instron Inc., Norwood, MA, USA) capable of generating controlled alternating motion in terms of displacement amplitude and frequency. The arm free end was connected to the actuator, while the fixed end was rigidly constrained using a steel yoke, as shown in Figure 4b.

**Figure 5:**
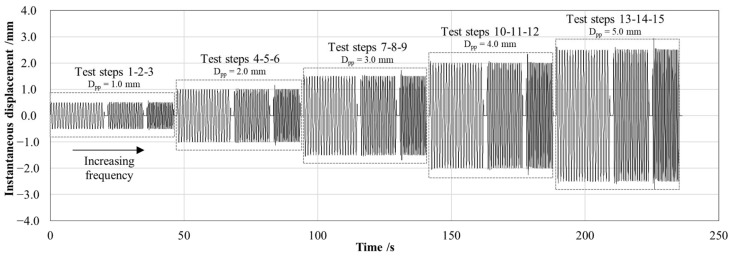
Displacement trend for electromechanical characterization of the arm–PCP system. Each test step was characterized by a combination of imposed peak-to-peak displacement (1.0, 2.0, 3.0, 4.0, 5.0 mm) and forcing frequency (1.0, 1.5, 2.0 Hz) to simulate the sea waves-induced pitching motion of the buoy. Each test step was performed for 20 cycles.

As shown in Figure 5, the system was subjected to cyclic loading under displacement control, applying a sinusoidal forcing function with different frequencies and amplitudes to replicate the pitching motion of the buoy induced by wave activity. A total of 15 test steps, each composed of 20 sinusoidal cycles, were performed by varying the combination of imposed peak-to-peak displacement (*D_pp_* equal to 1.0, 2.0, 3.0, 4.0, 5.0 mm) and forcing frequency (*f* equal to 1.0, 1.5, 2.0 Hz). The variable values were selected as representative of the typical nearshore sea wave conditions [30]. In addition, the maximum peak-to-peak displacement amplitude (*D_pp_* equal to 5.0 mm) ensures that the resulting load on the arm matches the predicted force obtained by the simplified mathematical model (≈24 N), as predicted by the FEM analysis. These experimental ranges, aligned with the theoretically expected working conditions, allow to guarantee the structural reliability of the system. Indeed, the PCP–arm bonding can withstand a maximum free end vertical displacement of up to 10 mm, at which the S-shaped arm remains in the linear elastic field.

The working conditions of the electrodynamic testing machine were acquired through the WaveMatrix3 software (Instron Inc., Norwood, MA, USA) in terms of the instantaneous digital position to retrieve the imposed vertical displacement of the S-shaped arm free end at a frequency of 100 Hz.

During the cyclic motion, the instantaneous voltages generated by the PCPs over a resistive load were acquired using the high-precision data acquisition system Sirius X (Dewesoft, Trbovlje, Slovenia) with a frequency of 1000 Hz. As the two PCPs behave in phase opposition, two separate channels were acquired. For each cycle (i.e., 1 sinusoidal displacement), the peak-to-peak generated voltage was then extracted (*V_pp,C_*), and for each test step (i.e., 20 sinusoidal cycles), the average peak-to-peak generated voltage (*V_pp,TS_*) was calculated.

From the generated peak-to-peak voltage, the effective power (*P_p,TS_*) was calculated under different forcing conditions, such as:

(3) $$P_{p,TS}=\frac{V_{pp,TS}^{2}}{4\cdot R_{opt}}$$

where *R_opt_* = 100 kΩ is the connected resistive load, optimized to maximize the power transfer [20].

A series of 18 measurements was conducted under comparable conditions in order to calculate the overall averaged peak-to-peak generated voltage (*V_pp_*) and effective power (*P_p_*) and estimate the measurement uncertainty.

Due to the irregular 3D shape of the arm and considering the presence of some geometrical peculiarities (i.e., curves, changes in thickness with the ribs, …), an open-field analysis through 3D visual scanning was performed to evaluate the geometric accuracy of the fabricated arms relative to the original CAD model used for stereolithographic printing. An optical scanner (model ATOS Compact Scan 2M, GOM) equipped with a structured blue light projector (400–500 nm, focal length of 12 mm) and two stereo cameras (focal length of 17 mm, resolution of 1624 × 1236 px^2^) positioned at a 25° angle was employed. To obtain a coherent three-dimensional model, 4 scans were acquired at different angles with respect to the sample (90° step) over a volume of 250 × 190 × 190 mm^3^ through the acquisition of 2 × 10^6^ points. The resulting three-dimensional model and point clouds were generated from the scans using a combination of manual and automated alignment procedures. The first manual alignment step was performed using four pairs of homologous markers, followed by the second automated step employing the iterative closest point (ICP) algorithm to achieve the final three-dimensional superposition. The geometric accuracy of the fabricated specimens was investigated through a shift analysis between the acquired point cloud and the reference CAD model using the open-source software CloudCompare.

## 3. Results

### 3.1. Electromechanical Characterization

**Figure 6:**
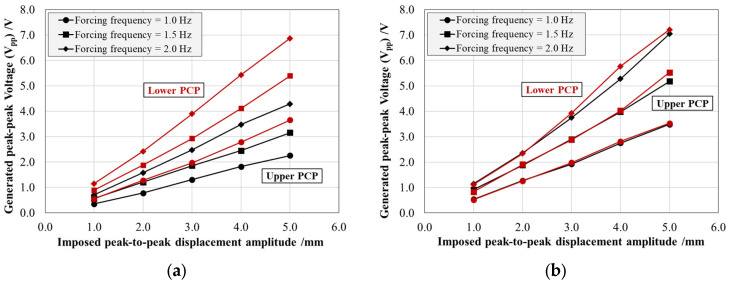
Peak-to-peak electric voltage (*V_pp_*) generated by the upper (black) and lower (red) PCPs as a function of the imposed peak-to-peak displacement amplitude simulating buoy pitching, at different forcing frequencies for the arm produced with the Black V4 (**a**) and Rigid 4000 (**b**) resins.

**Figure 7:**
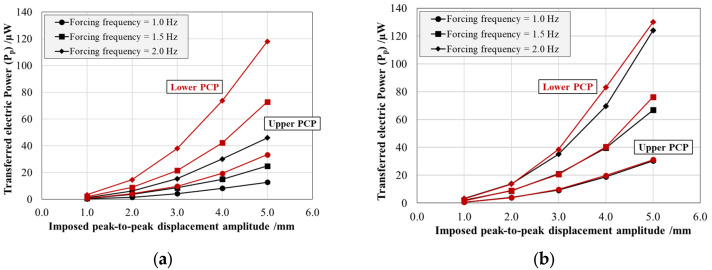
Effective power (*P_p_*) transferred by the upper (black) and lower (red) PCPs over the optimal electrical load of 100 kΩ as a function of the imposed peak-to-peak displacement amplitude simulating buoy pitching at different forcing frequencies for the arm produced with the Black V4 (**a**) and Rigid 4000 (**b**) resins.

Figure 6 and Figure 7 present the energy harvesting system response in terms of the average peak-to-peak electric voltage (*V_pp_*) generated and the average effective power (*P_p_*) transferred by the individual PCPs, each connected to an optimal resistive load of 100 kΩ. For both figures, the results are reported as a function of the imposed maximum peak-to-peak displacement amplitude and parameterized by the forcing frequency for both production resins: Black V4 on the left (a) and Rigid 4000 on the right (b). The black curves correspond to the patch bonded to the top surface of the arm, while the red curves represent the patch bonded to the bottom surface.

The generated peak-to-peak voltage (*V_pp_*) shows an approximately linear relationship with both displacement amplitude and forcing frequency, in accordance with theoretical expectations [31]. The increase in voltage with imposed displacement amplitude is a direct consequence of the piezoelectric effect as the accumulated electric charge is directly related to the intrinsic characteristic of the material (charge coefficient *d_31_*) and to the imposed strain. Similarly, at a constant displacement amplitude, the voltage output increases along with the forcing frequency. This is due to the fact that piezoceramic materials respond to the rate of change of deformation, meaning that higher frequencies result in a more rapid variation of strain over time, thereby enhancing charge generation and increasing voltage. However, this frequency-dependent increase is limited by the intrinsic capacitance of the piezoceramic material and the characteristics of the external resistive load, which can introduce saturation effects at sufficiently high frequencies.

In addition, while the Rigid 4000 system displays a symmetric behavior between the upper and lower patches, the Black V4 system exhibits a pronounced asymmetry, with a voltage ratio of approximately 64% between the two patches. This discrepancy can be attributed to two main factors: (i) variations in the mechanical response of the structure, mainly due to residual deformations post-AM process; (ii) imperfect adhesive bonding conditions, mainly causing a PCP position shift with respect to the optimal housing area and the bonding glue not homogeneously distributed between the patch and the resin. A similar pattern is observed in the effective power (*Pp*), which follows an exponential trend consistent with theoretical predictions.

**Figure 8:**
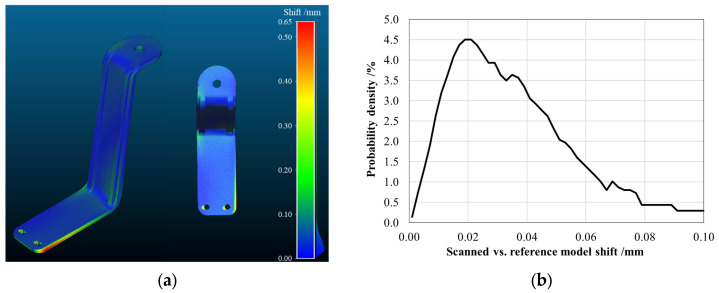
Quality control of a Black V4 resin-type arm. (**a**) Absolute shift map between the 3D scan acquisition and the reference CAD model with the global (left) and top (right) views. (**b**) Probability density function for the rigid shift due to the acquired and reference 3D model alignment processes.

Considering the highlighted results, the S-shaped arm manufacturing quality control was performed. Figure 8a shows the absolute shift maps obtained by comparing the point cloud acquired using the 3D scan with the reference 3D CAD model used as input for the stereolithographic printing process, in the case of the arm manufactured with the Black V4 resin. In an ideal scenario, this map should exhibit a zero shift, indicating a perfect match between the designed model and the fabricated component. However, as reported in Figure 8b, the analysis reveals a slight overall shift in the order of 0.020 ± 0.005 mm, which can be attributed to the inherent alignment process of the point clouds, therefore, not jeopardizing the accuracy of the fabrication process.

Conversely, a more pronounced distance is observed at the fixed end of the arm (the lower region in the figure), specifically at the PCP housing area, where the shift reaches the peak value of 0.65 ± 0.10 mm. This localized distance can be associated with torsional deformation occurring during the printing and curing stages, likely induced by differential shrinkage and internal stress accumulation within the polymeric structure. This residual deformation could affect the structural response of the S-shaped arm, leading to a different intrados/extrados behavior during the harvesting process with a consequent asymmetry in voltage generation.

The presence of this deformation was consistently detected across all the tested specimens made with the Black V4 resin, suggesting that it is an inherent characteristic of the manufacturing process, especially as compared with all the curing conditions, which should be optimized.

### 3.2. Measurement Uncertainty Evaluation

The measurement uncertainty was evaluated based on the methodologies reported in the Guide to the Expression of Uncertainty in Measurement (GUM) [32]. For the peak-to-peak electric voltage (*V_pp_*), a type A measurement uncertainty was calculated using direct experimental data from the 18 tests performed. For the optimal electrical load (*R_opt_*), the nominal value was estimated by means of a digital multimeter with an accuracy of 0.1 kΩ, therefore, a type B measurement uncertainty was defined considering a uniform probability density function. For the effective power (*P_p_*), the combined uncertainty was estimated by considering the relative weight of each variable in Equation (3) and applying the standard uncertainty propagation law:

(4) $$U_{Pp}=\sqrt{\sum_{i}u^{2}\left(x_{i}\right)\cdot {\left(\frac{\delta \bar{P_{p}}}{\delta x_{i}}\right)}^{2}}={\left[u^{2}\left(V_{pp}\right)\cdot {\left(\frac{\delta \bar{P_{p}}}{\delta V_{pp}}\right)}^{2}+u^{2}\left(R_{opt}\right)\cdot {\left(\frac{\delta \bar{P_{p}}}{\delta R_{opt}}\right)}^{2}\right]}^{0.5}$$

where *u(V_pp_)* and *u(R_opt_)* represent the uncertainties in the measured peak-to-peak voltage and the resistive optimal load, respectively. The extended uncertainty (*U_Pp_*) was estimated assuming a coverage factor (*k*) of 2.1098, corresponding to a t-Student distribution with 17 degrees of freedom and a 95% confidence level. The detailed computation is shown in Table 2.

Analyzing the relative contributions to the uncertainty, it can be highlighted that the dominant factor was related to the voltage measurement, whereas the uncertainty associated with the resistive load was quite negligible. Similar uncertainty levels were observed across all the different loading conditions, leading to an extended uncertainty of the order of 0.82% for the generated peak-to-peak electric voltage (*V_pp_*) and 1.65% for the effective power (*P_p_*) transferred, relative to their respective mean values.

Comparing the response of the two energy harvesters produced with different resins, it is obvious that the system behavior is strongly influenced by the asymmetry observed in the Black V4 patches. However, focusing on the lower patch alone, the two materials exhibited a highly comparable performance, with variations in the peak-to-peak voltage (*V_pp_*) of ≈1% and in the effective power (*P_p_*) of ≈2%, both within the estimated measurement uncertainty range. This effect can be attributed to the testing methodology, which employed displacement-controlled rather than force-controlled oscillation.

### 3.3. Electrical Connection of the Patches

**Figure 9:**
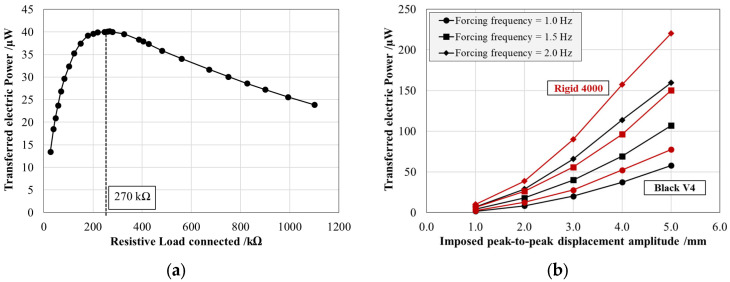
(**a**) Definition of the optimal resistive load for the Black V4 energy harvester in the case of an imposed peak-to-peak displacement of 3 mm and a forcing frequency of 1.5 Hz. (**b**) Effective power transferred by the two PCPs connected in parallel with inverted poles over an optimal electrical load of 270 kΩ as a function of the imposed peak-to-peak displacement amplitude simulating buoy pitching at different forcing frequencies for the arm produced with the Black V4 (black) and Rigid 4000 (red) resins.

As described in the dedicated section, the PCPs were installed on opposite faces of the patch housing. As a consequence, during the sinusoidal loading, they experienced mechanical deformations with opposite signs, resulting in a generated electrical voltage phase shift of 180°. To optimize the generated electrical output, the PCP outputs were connected through a parallel electrical configuration with inverted poles. Figure 9a shows the effective power output (*P_p_*) as a function of the resistive load for the Black V4 system at a mechanical loading frequency of 1.5 Hz and a displacement amplitude of 3 mm. With the adopted connection, the resistive load that allows for the maximization of effective power transfer results in a value equal to 270 kΩ.

Figure 9b presents the average effective power output for the PCPs connected in parallel with inverted poles at the optimal 270 kΩ resistive load as a function of the displacement amplitude and the forcing frequency for both produced resins. The results confirm that the effective power output significantly exceeded that of an individual patch, validating the effectiveness of the chosen electrical configuration. For instance, under sinusoidal excitation at 1.5 Hz with a 3 mm peak-to-peak displacement, the transferred effective power reached (40.0 ± 0.7) µW for the Black V4 system and (55.7 ± 0.9) µW for the Rigid 4000 system.

## 4. Discussion and Conclusions

The present study investigated the development and characterization of an energy harvesting system based on PCPs integrated into a wave-driven motion conversion arm. The proposed system was studied using FEM analysis to optimize the load transfer in the PCPs housing section by enhancing the S-shaped arm vertical stiffness and geometrical design parameters. It was fabricated via stereolithographic additive manufacturing and subsequently tested for electromechanical performance evaluation and production quality control.

The experimental results demonstrated the systems capability to generate voltage and transfer effective power under cyclic loading conditions typical of nearshore applications, confirming the effectiveness of the proposed solution. Furthermore, the electrical response of the PCPs aligned with theoretical predictions regarding the relationship between the displacement amplitude, the forcing frequency, and the generated voltage, validating the energy harvesting mechanical design. The maximum transferred electric power of 130.0 ± 2.2 µW was reached at a 5.0 mm displacement amplitude and a 2 Hz sinusoidal sea wave, with the generated *V_pp_* of 7.21 ± 0.06. Additionally, implementing an optimized parallel electrical connection with inverted poles between the patches significantly enhanced the overall power transfer efficiency with maximum 220.2 ± 3.7 µW at a 5.0 mm displacement amplitude and a 2 Hz frequency, making the system more suitable for practical deployment.

The proposed system, although not operating under mechanical resonance conditions, and consequently resulting in lower power harvesting, is able to convert the continuous 24 h per day kinetic energy of the sea wave. Unlike other energy harvesting systems in the literature, the reduced overall dimensions of the proposed device allow for easy installation in a pre-existing buoy concept. Finally, the system’s modularity facilitates straightforward scaling in accordance with the desired electrical power output and the dimensions of the same buoy.

However, several challenges emerged during the production phase. While the overall dimensional accuracy remained within the acceptable tolerances (deviation of 0.020 ± 0.005 mm compared to the reference CAD shape), residual localized deformations near the PCP housing were detected (a 0.65 ± 0.10 mm shift compared to the reference CAD shape). These deformations, primarily due to differential shrinkage and internal stress accumulation during the curing process, may affect strain transmission and should be mitigated through improved curing protocols. In addition, the manual adhesive bonding of the PCPs is an operator-dependent process, and eventual inaccuracies could jeopardize the energy harvesting performance. All these manufacturing-induced issues highlight the need for improved post-processing techniques and more precise installation methods.

Regarding the material selection, the comparative evaluation of the Black V4 and Rigid 4000 resins revealed similar energy conversion capabilities when considering the lower patch alone under the displacement-controlled test conditions (at 1.5 Hz with a 3 mm peak-to-peak displacement of 40.0 ± 0.7 µW for the Black V4 system and of 55.7 ± 0.9 µW for the Rigid 4000 system). However, in a real operating scenario where the transferred force is the main governing parameter, the higher stiffness of the Rigid 4000 resin would lead to a reduced structural deformation compared to the Black V4 resin, potentially reducing the energy conversion capability. Despite this, the superior mechanical stability of Rigid 4000 makes it a more reliable choice for practical applications to increase system durability.

The next phase of this research will involve the installation of the characterized prototype on a monitoring buoy [33] within a marine environment to assess its long-term performance under realistic sea conditions. Obviously, in real applications, downstream of the energy harvester, a conversion electronics module must be connected to allow for the generated electrical power storage. A rectification and regulation circuit (RRC) should be used to convert the AC output into a DC signal [20], useful as a power source to feed a storage device connected to a low-voltage electric load (e.g., remote wireless sensors). The acquired data will be crucial for validating the system energy harvesting potential and identifying further improvements. Future work will focus on enhancing both the mechanical and electrical design, exploring alternative production techniques and optimizing post-processing protocols to minimize geometric deviations.

## Figures and Tables

**Table 1:** Mechanical characteristics of photopolymer resins Black V4 and Rigid 4000 [28].

Resin	Density @ 25 °C, g/cm^3^	Ultimate Tensile Strength, MPa	Tensile Modulus, GPa	Elongation at Break, %	Flexural Modulus, GPa	Notched Izod, J/m
Black V4	1.09	65	2.8	6.0	2.2	25
Rigid 4000	1.26	69	4.1	5.3	3.4	23

**Table 2:** Extended uncertainty calculation details in the case of loading frequency of 1.5 Hz and displacement amplitude of 1 mm.

Parameter	Mean Value	Uncertainty Type	u(x_i_)	u^2^∙(δP/δx_i_)^2^	u(P_p_), µW	U_c_(P_p_), µW	U_c_(P_p_)/P_p_, %
V_pp_/V	0.80	A	3.0 × 10^−3^	1.7 × 10^−16^	1.3 × 10^−2^	2.7 × 10^−2^	1.7%
R/Ω	1× 10^5^	B	5.8 × 10^1^	9.9 × 10^−19^			

## Data Availability

Data are contained within the article.

## Abbreviations

The following abbreviations are used in this manuscript:

PZT	Piezoelectric
PCPs	Piezoceramic patches
FEM	Finite element model
CAD	Computer-aided design
ICP	Iterative closest point
ISO	International Organization for Standardization
IEC	International Electrotechnical Commission
